# Retention of fluoride in the oral cavity: A randomized clinical study with nanoencapsulated fluoride toothpaste

**DOI:** 10.4317/jced.63567

**Published:** 2026-03-30

**Authors:** Marcel Alves Avelino de Paiva, Helene Soares Moura, Matheus Barbosa de Medeiros Souza, Alexandre Almeida Júnior, Andressa Feitosa Bezerra de Oliveira, Fábio Correia Sampaio

**Affiliations:** 1 Department of Clinical and Social Dentistry, Federal University of Paraiba, João Pessoa, Paraiba, Brazil; 2 Department of Dentistry, State University of Paraíba (UEPB), Araruna, Paraíba, Brazil; 3 Department of Morphology, Federal University of Paraiba, João Pessoa, Paraiba, Brazil

## Abstract

**Background:**

To evaluate in vivo fluoride retention in saliva and dental biofilm after the use of experimental toothpaste containing nanoencapsulated fluoride (NanoF).

**Material and Methods:**

A triple-blind randomized clinical study involving sixty-one residents from a municipality without fluoridated drinking water. The toothpastes used in the study were: Group 1 (1100 ppm, 100% NanoF); Group 2 (1100 ppm, 50% free NaF + 50% NanoF); and Group 3 (1100 ppm, 100% free NaF). During the use of the toothpaste, samples of dental biofilm and stimulated saliva were collected. Collection times were 1 and 2 hours (h) post-brushing for biofilm, and 1, 5, 10, 15, 20, 30, 45, 60 minutes, and 2h for saliva. The fluoride [F] concentrations in saliva and dental biofilm were analyzed using a specific electrode through the hexamethyldisiloxane (HMDS) facilitated diffusion technique. The data were analyzed using the Kruskal-Wallis, Friedman, and Wilcoxon tests (p&lt;0.05).

**Results:**

The values of the area under the curve (AUC) for saliva over 0-60 minutes were: G3 (97.74) &gt; G2 (84.73) &gt; G1 (54.98). For dental biofilm, the highest fluoride [F] concentration was observed in G1 (35.26 mg/kg), followed by G3 (34.06 mg/kg) at the 1-hour mark; at 2 hours, the results were 24.88 mg/kg and 31.43 mg/kg, respectively, with a significant reduction in [F] observed only in G1 (p=0.02).

**Conclusions:**

The nanoencapsulated fluoride tested in this study exhibited a distinct pattern of fluoride release in saliva. However, in dental biofilm, the release pattern was similar to that of the free fluoride formulation (NaF).

## Introduction

Dental caries results from the interaction between dental structure and acids produced by bacterial metabolism, leading to enamel demineralization. The recurrent imbalance between dental minerals and the dental biofilm fluid can result in more intense demineralization (1). The dynamics of enamel demineralization and remineralization occur constantly in the oral environment; however, when the risk factors for demineralization intensify, dental substance loss occurs (2). Preventive interventions against dental caries based on fluoride, particularly toothpastes, represent the most significant and widely disseminated approach for caries control globally (3 , 4). However, formulating effective fluoride-containing toothpaste is not straightforward. Muhler et al. (5) reported the first successful trial, which was followed by a deluge of reports. These reports led to the acceptance of these toothpastes, and today, health authorities in various countries and international organizations, such as the World Health Organization (WHO) and International Dental Federation (FDI), promote their use (4). Since the 1970s, fluoride toothpaste has been the primary factor associated with the reduction of caries prevalence observed in many countries (3). Its effectiveness in enamel remineralization is well-established in literature, acting to form a calcium fluoride layer on the dental surface and slowing the demineralization process (6 - 8). When present in the oral environment, fluoride reduces the demineralization of dental enamel, and this effect is directly related to its concentration (9). However, increasing the substantivity of fluoride remains a significant challenge, as the driving force of salivary clearance exceeds the strategies developed to enhance its retention in the oral cavity and thus reduce the prevalence of dental caries (10 , 11). In this regard, to prolong the bioavailability of substances and enhance their therapeutic efficacy, drugs are conjugated with various materials, resulting in the creation of advanced nanodrug delivery systems (12 - 14). Thus, the development of formulations that enable controlled fluoride release has garnered significant attention, as they meet the need for enhanced retention and controlled release on the dental surface, aiming to maintain therapeutic concentrations, promote the prevention of demineralization, and facilitate remineralization (6 , 15). Therefore, the present study aims to evaluate, in vivo, the retention of fluoride in saliva and dental biofilm after the use of experimental toothpastes containing nanoencapsulated fluoride (NanoF). The hypothesis being tested is that NanoF would be able to significantly increase the fluoride [F] concentration in both biofilm and saliva, even 2 hours after tooth brushing, compared to conventional toothpaste.

## Materials and Methods

**Figure 1:**
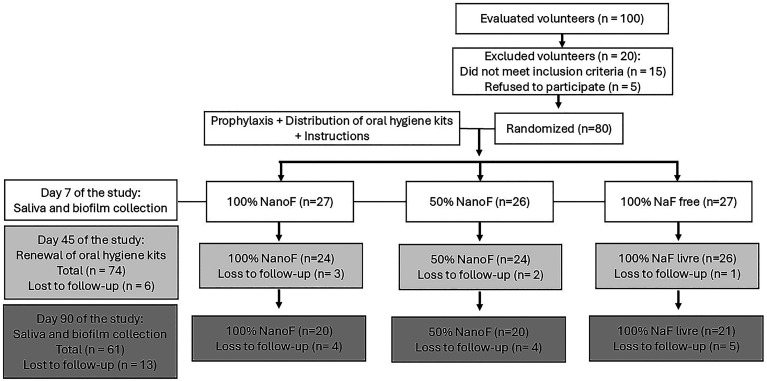
CONSORT flowchart of the study.

1. Study Type A crossover randomized triple-blind trial was designed to evaluate the bioavailability of intraoral fluoride in biomarkers of exposure (biofilm and saliva) after use of experimental fluoride dentifrices (n = 3) over the course of 1 week with washout periods in between uses. Biofilm (primary variable) was collected at 1 and 12 h after toothbrushing, and saliva was collected (secondary variable) at 1-h (t1, t5, t10, t15, t20, t30, t45, and t60 min) and 12-h periods. Potentiometry (ion-selective electrode) was employed for fluoride analysis of these samples. A pilot test was performed with three participants prior to the start of the study. 2. Sample The sample size was calculated based on previous studies (13 , 16), considering a significance level of 5% ( = 0.05), a statistical power of 90% ( = 0.10), and an anticipated dropout rate of 10%. The calculation indicated the need for approximately twenty-five participants per group. However, due to operational limitations inherent to the conduct of the clinical study, including participant losses during follow-up, the final sample consisted of sixty-one children of both sexes, aged 12.1 (1.60) years, were divided into three groups: G1) twenty participants, G2) twenty participants, and G3) twenty-one participants. All children were residents of the metropolitan area of João Pessoa, Paraíba (&lt;0.01 g/mL F, an area without a water fluoridation program). The use of orthodontic appliances, treatment with fluoride products, use of antimicrobials in the last 4 weeks, presence of cavitated carious lesions, periodontal disease, and dental sensitivity were considered exclusion criteria. 3. Dentifrices and Group Allocation Participants were randomly assigned to one of three dentifrice groups: G1 (100% NanoF): Dentifrice containing 100% nanoencapsulated sodium fluoride (1100 ppm F-); G2 (50% NanoF): Dentifrice containing a 50:50 mixture of nanoencapsulated fluoride and free sodium fluoride (1100 ppm F-); G3 (100% NaF): Dentifrice containing 100% free sodium fluoride (1100 ppm F-). The dentifrices were prepared, coded, and packaged by Savoy SA (São Paulo, Brazil), which was not involved in participant recruitment, data collection, or analysis. Each formulation was provided in identical unlabeled tubes, identified only by a color code: yellow, green, or blue. The correspondence between colors and formulations was kept confidential by the company and disclosed to the researchers only after the completion of all experimental procedures and statistical analyses. All dentifrices were similar in color, flavor, and composition, differing only in the fluoride delivery system. Group allocation was performed using a lottery system with colored cards representing each coded dentifrice. The random sequence was generated by thoroughly shuffling the cards before each draw, ensuring equal allocation probability. The cards were enclosed in opaque envelopes and opened sequentially after participant enrollment to maintain allocation concealment. Both participants and investigators responsible for data collection and outcome assessment were blinded to group assignment. The coded samples were maintained throughout all experimental stages, ensuring that examiners remained unaware of the treatment group and evaluation time point until the study was fully completed. 4. Clinical Procedures First, the participants underwent dental prophylaxis to remove all biofilm and dental calculus and used a placebo dentifrice for 7 days (lead-in period). We followed the protocols of Whitford et al. (17 , 18) and Pessan et al. (19 , 20). Subsequently, all participants used the toothpastes (100% NanoF, 50% NanoF, and 100% free NaF) three times a day for ninety days. They were instructed to brush only the occlusal surfaces on the sixth day of use and not to use dental floss to allow biofilm accumulation on the smooth surfaces. The following day, 2 hours after the last brushing, saliva and dental biofilm samples were collected (upper and lower right hemi-arch). Next, the volunteers brushed only the occlusal surfaces again for 1 minute. Following this, saliva samples were collected at the following times: t1, t5, t10, t15, t20, t30, t45, and t60 minutes. One hour after brushing, dental biofilm samples (left side) were collected. Stimulated saliva (by chewing silicone) was collected for 2 minutes. The dental biofilm was collected from all smooth surfaces of the teeth (buccal and lingual) using a Hollenback 3S spatula (Millennium - Golgran), after which the samples were immediately transferred to an Eppendorf tube, centrifuged, weighed, and subsequently dried at 90 °C for two hours; they were then reweighed and stored at -20 °C until analysis. The saliva samples were centrifuged at 6,000 rpm for 10 minutes to separate/remove the supernatant. At the end of the study, after 90 days of using the toothpastes, the procedure for collecting saliva and dental biofilm was repeated. The CONSORT diagram of the study (recruitment, intervention allocation, follow-up) is presented in Figure 1.

1

**Figure 2:**
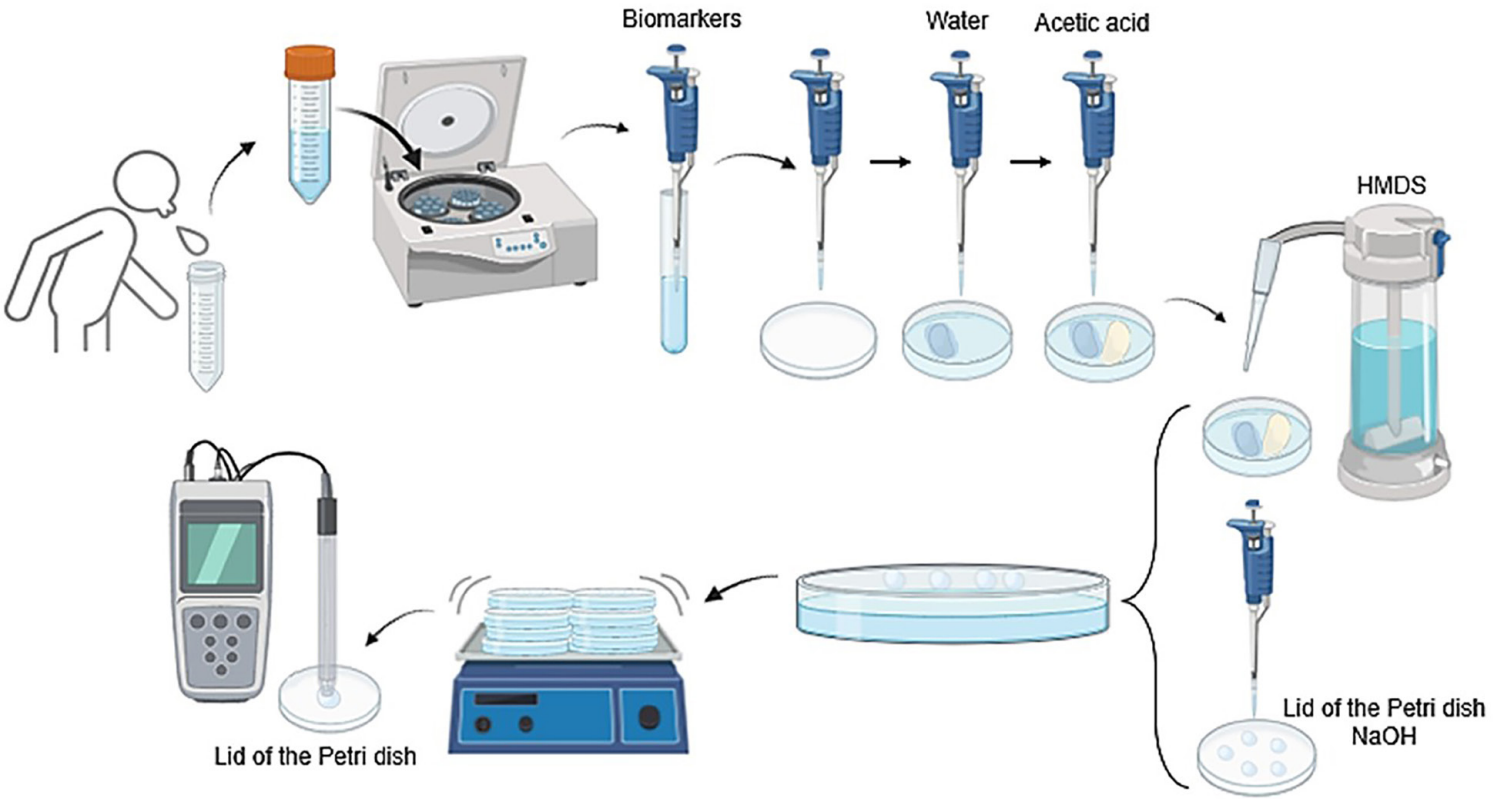
Illustration of the facilitated diffusion technique using HMDS (Hexamethyldisilazane).

5. Determination of Fluoride Concentration The samples were analyzed using the facilitated diffusion method with hexamethyldisiloxane (HMDS) as modified by Taves (21) and previously applied in similar studies (11) (Fig. 2).

2

The analyses were performed in triplicate using a fluoride-specific electrode (model 9409; Orion Research) and a potentiometer (model EA 940; Orion Research, Cambridge, MA, USA) (Model 720 A Orion). Fluoride standards (1-100 nM) were used to prepare the calibration curves. 6. Statistical Analysis The Shapiro-Wilk test was applied to assess the normality of the data. Comparisons of fluoride concentrations in saliva and dental biofilm between groups were performed using Kruskal-Walli's test. For comparisons among individuals within the same group, the Friedman and Wilcoxon tests were used (p &lt; 0.05). Effect sizes were calculated for both tests: Kendall's W was used to assess the magnitude of time-related differences in repeated measures (Friedman test), and the effect size r was calculated for pairwise comparisons (Wilcoxon test). The area under the curve (AUC) was calculated to indicate the effectiveness of fluoride retention in saliva over time, considering fluoride values at the time interval from 2 hours to 60 minutes after the last brushing. Data were calculated using Graph Pad Prism (version 6) and the Statistical Package for the Social Sciences (SPSS) version 20.

## Results

**Figure 3:**
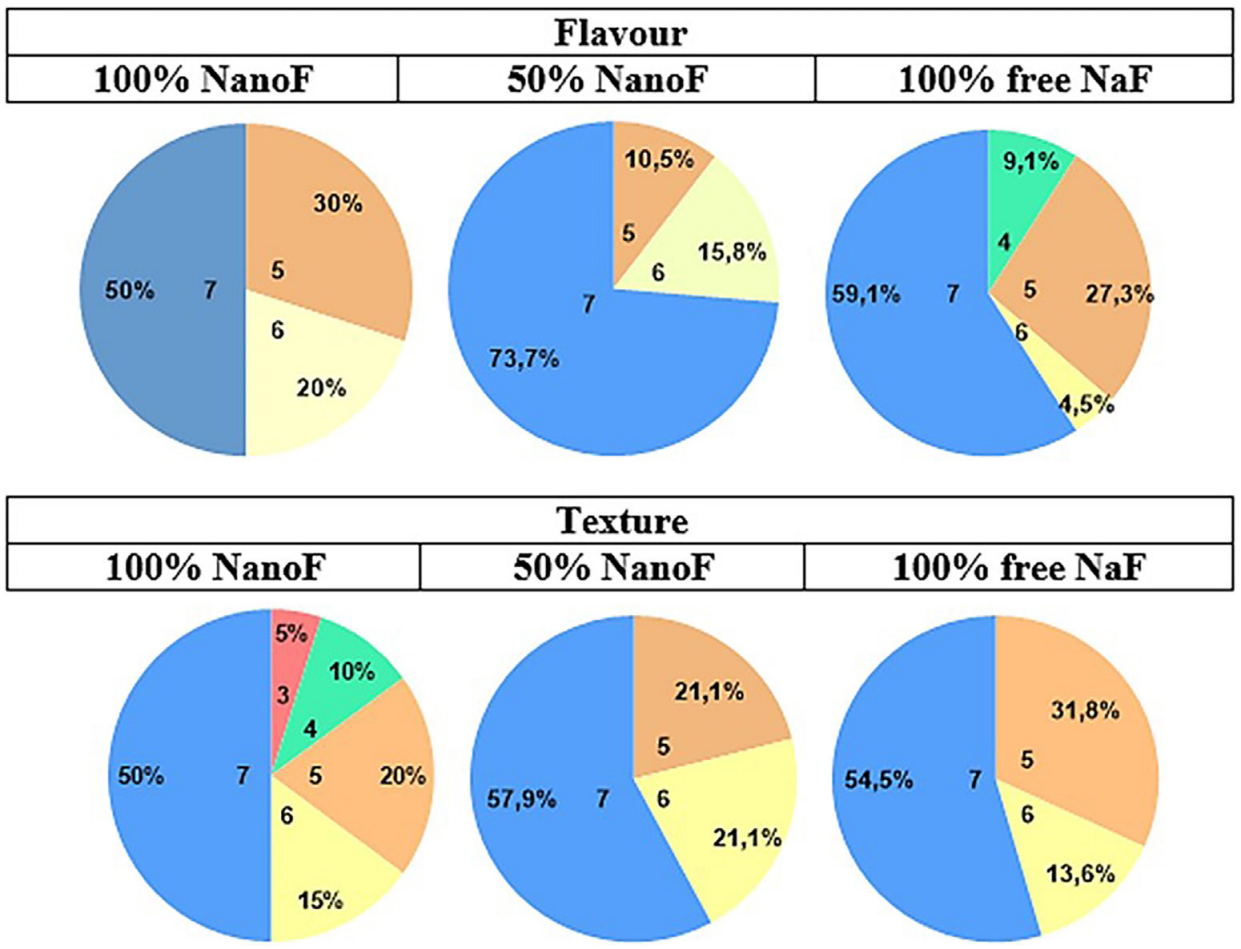
Acceptability of toothpastes (flavor and texture) where 0 is very dissatisfied (the worst possible flavor/texture) and 7 is excellent (the best possible flavor/texture).

The compliance of the volunteers to the study was considered satisfactory. The toothpastes were well accepted by the participants (Fig. 3).

3

Regarding the study regimen, most participants reported performing oral hygiene at least twice a day with the study toothpaste. Salivary [F] data were calculated from the AUC0-60min (Table 1).

Table 1

G3 (100% NaF) presented the highest fluoride concentration, followed by G2 (50% NanoF) and G1 (100% NanoF), but no significant difference was observed. All toothpaste exhibited a similar behavior over the long term. The highest fluoride concentration values were observed at the initial time point (t1), followed by a sharp decrease after 5 minutes of use (t5), a further reduction after 10 minutes (t10), and then they remained relatively stable, decreasing more slowly during the subsequent time points. The mean fluoride concentrations in saliva at different time intervals are detailed in Table 2.

Table 2

A significant difference was observed among the groups (t 20 e t 45). In the intragroup analysis, significant differences were also found over time (p &lt; 0.05), with effect size estimates indicating a strong influence of time on salivary fluoride levels in all groups: G1 (W = 0.951), G2 (W = 0.959), and G3 (W = 0.985). The values of fluoride concentrations retained in the dental biofilm collected 1 hour and 2 hours after the last brushing can be observed in Table 3.

Table 3

**Figure 4:**
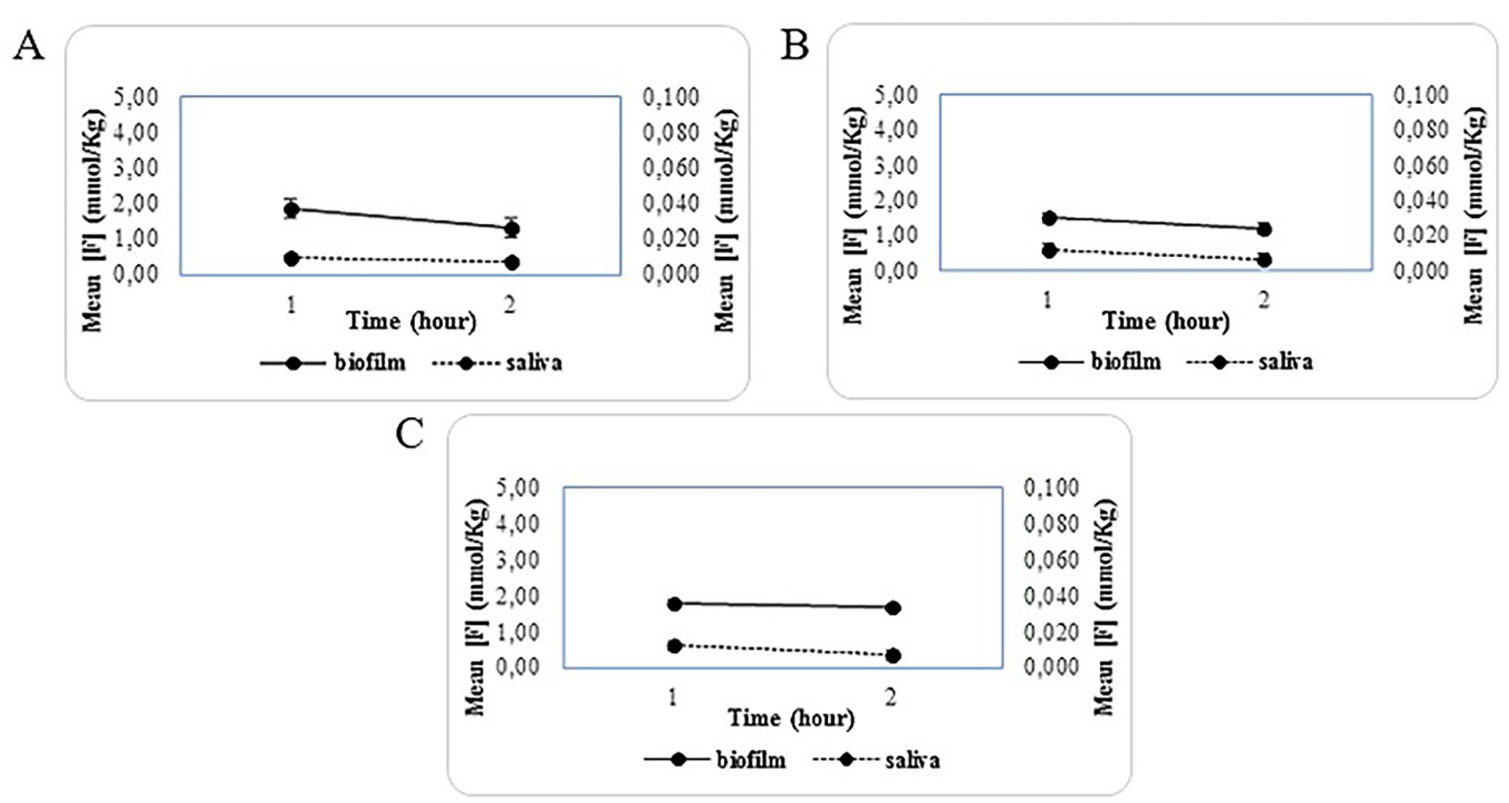
Relationship between [F] in dental biofilm and saliva after the use of toothpaste at 1 and 2 hours. A) G1 - 100% NanoF; B) G2 - 50% NanoF; C) G3 - 100% free NaF.

A significant difference was observed in group G1 (p = 0.02), with a moderate effect size (r = 0.38), indicating a relevant reduction in the fluoride concentration between 1 h and 2 h. No statistically significant differences were found in groups G2 and G3. Figure 4 show the interaction of fluoride present in the dental biofilm and saliva 1 hour and 2 hours after the use of the toothpastes employed in the study.

4

It is observed that all groups exhibited a reduction in [F] in saliva; however, group G1 (100% NanoF) showed values trending toward equilibrium. As for the dental biofilm, group G1 (100% NanoF) exhibited the highest [F] at 1 hour after brushing; however, over the 2-hour period, all groups behaved similarly, showing a reduction in [F].

## Discussion

This study evaluated a nanoparticle system composed of bioadhesive polymers that release fluoride ions more slowly, consisting of sodium fluoride (NaF) encapsulated in a natural biopolymer. In this system, fluoride release does not depend on pH but rather on enzymatic triggers following a controlled release mechanism. The nanoparticles undergo biodegradation by salivary amylase, allowing for a more efficient release of fluoride in the oral environment while preventing rapid clearance by saliva (6 , 22). The composition of NanoF is primarily sodium fluoride (NaF) encapsulated in a natural biopolymer (hydroxypropyl guar). Guar gum is a carbohydrate polymer of molecular weight derived from the natural seed of the guar plant (Cyamopsis tetragonoloba (L.) Taub) (6). The study was conducted in a city that does not have water fluoridation, ensuring that the fluoridated toothpaste was the primary source of fluoride in the study. The hypothesis tested in this study assumed that there was a statistically significant difference between the fluoride bioavailability of G1 and G2 when compared with G3. The amount of fluoride used and the technique applied during brushing directly influence the retention of fluoride in biomarkers of oral exposure (23 , 24). In this study, the use of a transverse technique for dispensing toothpaste onto the toothbrush was recommended (23 , 25). The physiological process by which substances are removed from the oral cavity (salivary clearance) reduces the amount of fluoride over time in oral fluids (26). Therefore, an additional benefit of using a toothpaste containing nanoencapsulated fluoride would be the continuous release of this particle in the oral cavity, maintaining its ideal levels for an extended period and functioning as an additional reservoir of fluoride. Average fluoride concentration data (AUC) in saliva over time for the different evaluated toothpastes show that the conventional NaF-free toothpaste (G3) exhibited [F] levels in saliva higher than those of the experimental toothpaste (G1 and G2). The kinetics of fluoride in saliva showed a rapid decrease for all groups within the first 30 minutes after brushing, highlighting the effect of salivary clearance, consistent with previous findings (11 , 16). Despite the significant reduction in fluoride levels in G1 (100% NanoF), the concentration in saliva showed a tendency toward equilibrium at 1 and 2 hours, suggesting a feedback mechanism in the interaction between saliva and biofilm. Thus, the biofilm can retain fluoride for prolonged periods, contributing to the maintenance of intraoral fluoride levels, especially under cariogenic challenge conditions and with baseline fluoride values present (11). The nanoencapsulation of fluoride represents an advancement in this field, ensuring its release over prolonged periods without increasing the risk of fluorosis. Conversely, it exhibits a lower intrabucal fluoride concentration, primarily due to the influence of salivary clearance. However, to ascertain how these results may affect the control of dental caries, in vivo models are the most suitable for evaluating the influence of NanoF-containing toothpaste on the remineralization of active white spot lesions. Furthermore, it is essential to emphasize the complexity of formulating toothpaste, as it consists of a mixture of abrasives suspended in a hydrating aqueous phase, forming a hydrocolloid material in which the active ingredients are incorporated. Once in the oral cavity, this material forms a paste - a mixture of saliva and denser solids derived from the toothpaste. On one hand, saliva facilitates the dispersion of these ingredients throughout the mouth; on the other hand, it dilutes all components, including the bioactive substances. Ultimately, these bioactive ingredients must remain stable in various pH environments (27). Despite the increasing popularity of fluoride-free remineralization technologies, such as bioactive silica toothpaste described by Sampaio et al. (27) the presence of fluoride remains essential for the formation of a more acid-resistant layer (28). To the best of my knowledge, this is the first in vivo study to discuss the fluoride retention capacity in the oral cavity after the use of a toothpaste containing sodium fluoride (NaF) encapsulated in a natural biopolymer as a controlled release system. Furthermore, the significance of the relevant results obtained for the NanoF toothpaste is emphasized, as they may serve as a reference for future formulations aimed at gradual fluoride release. Individual variations in the release rate were not investigated but may exist, suggesting that the individual response could be modulated by intraoral factors in saliva and dental biofilm. Monitoring these biomarkers may indicate changes in intraoral fluoride levels and could reflect the anticariogenic efficacy of fluoridated products (28). One of the limitations of this study was the participant dropout rate, which reached 20%. This dropout can be attributed to factors such as changes of address and participants' scheduling conflicts, which may have impacted the final sample. However, the data collected still provide valuable insights into the investigated topic.

## Conclusions

The results of this study suggest that toothpaste with NanoF (G1 and G2) exhibited a different release pattern in saliva. In the dental biofilm, the release pattern was similar to that of the free fluoride formulation (NaF).

## Figures and Tables

**Table 1:** Area under the curve (AUC) of the mean fluoride concentration in saliva.

Toothpastes	AUC0-60
G1 (100% NanoF)	54.98
G2 (50% NanoF)	84.73
G3 (100% free NaF)	97.74
p-value*	0.36

*Kruskal-Wallis test

**Table 2:** Mean (SD) of fluoride concentration in saliva (mM) at different collection times.

Toothpastes	Time (min)								
	t1	t5	t10	t15	t20	t30	t45	t60	t120
G1 (100% NanoF)	0.519a (0.301)	0.107a (0.067)	0.039a (0.028)	0.024a (0.019)	0.018a (0.012)	0.014a (0.010)	0.012a (0.012)	0.009a (0.005)	0.006a (0.003)
G2 (50% NanoF)	0.823a (0.458)	0.185a (0.143)	0.068a (0.052)	0.037a (0.026)	0.025b (0.014)	0.018a (0.011)	0.014b (0.007)	0.011a (0.004)	0.006a (0.003)
G3 (100% free NaF)	0.951a (0.659)	0.251a (0.372)	0.093a (0.124)	0.051a (0.060)	0.031a,b (0.026)	0.019a (0.011)	0.017b (0.010)	0.012a (0.006)	0.007a (0.005)

Different lowercase letters in the same column indicate statistical differences, Kruskal-Wallis test. Highlighted cells indicate intragroup comparisons with means (SD) significantly different from their values at the final time point (t120), Friedman test (p < 0.05).

**Table 3:** Mean (SD) of fluoride concentration in dental biofilm (mg/kg) at different collection times.

Toothpastes	N	Time (Hour)		p-value*
		1h	2h	
G1 (100% NanoF)	20	35.26 (20.18)A	24.88 (13.76)B	0.02
G2 (50% NanoF)	20	28.42 (19.21)A	22.80 (14.03)A	0.22
G3 (100% free NaF)	21	34.06 (23.63)A	31.43 (25.08)A	0.07

* Wilcoxon test; different uppercase letters in the same row indicate statistical differences (p < 0.05). There is no difference between the groups, Kruskal-Wallis test.

## Data Availability

The data that support the findings of this study are available from the corresponding author upon reasonable request.
